# Genetic Polymorphisms Influence the Ovarian Response to rFSH Stimulation in Patients Undergoing In Vitro Fertilization Programs with ICSI

**DOI:** 10.1371/journal.pone.0038700

**Published:** 2012-06-11

**Authors:** Radia Boudjenah, Denise Molina-Gomes, Antoine Torre, Marianne Bergere, Marc Bailly, Florence Boitrelle, Stéphane Taieb, Robert Wainer, Mohamed Benahmed, Philippe de Mazancourt, Jacqueline Selva, François Vialard

**Affiliations:** 1 Department of Reproductive Biology, Cytogenetics, Gynaecology and Obstetrics, Poissy Saint Germain Medical Centre, Poissy, France; 2 EA 2493, Versailles Saint Quentin University, Versailles, France; 3 Department of Reproductive Biology, Nice University Hospital, Nice, France; Clermont Université, France

## Abstract

**Introduction:**

Obtaining an adequate number of high-quality oocytes is a major challenge in controlled ovarian hyperstimulation (COH). To date, a range of hormonal and clinical parameters have been used to optimize COH but none have significant predictive value. This variability could be due to the genetic predispositions of single-nucleotide polymorphisms (SNPs). Here, we assessed the individual and combined impacts of thirteen SNPs that reportedly influence the outcome of *in vitro* fertilisation (IVF) on the ovarian response to rFSH stimulation for patients undergoing intracytoplasmic sperm injection program (ICSI).

**Results:**

Univariate analysis revealed that only FSHR, ESR2 and p53 SNPs influenced the number of mature oocytes. The association was statistically significant for FSHR (p=0.0047) and ESR2 (0.0017) in the overall study population and for FSHR (p=0.0009) and p53 (p=0.0048) in subgroup that was more homogeneous in terms of clinical variables. After Bonferroni correction and a multivariate analysis, only the differences for FSHR and ESR2 polymorphisms were still statistically significant. In a multilocus analysis, only the FSHR and AMH SNP combination significantly influenced oocyte numbers in both population (p<0.01).

**Discussion:**

We confirmed the impact of FSHR and ESR2 polymorphisms on the IVF outcome. Furthermore, we showed for the first time that a p53 polymorphism (which is already known to impact embryo implantation) could influence the ovarian response. However, given that this result lost its statistical significance after multivariate analysis, more data are needed to draw firm conclusions. Only the FSHR and AMH polymorphism combination appears to influence mature oocyte numbers but this finding also needs to be confirmed.

**Materials and Methods:**

A 13 gene polymorphisms: FSHR(Asn680Ser), p53(Arg72Pro), AMH(Ile49Ser), ESR2(+1730G>A), ESR1(−397T>C), BMP15(−9C>G), MTHFR1(677C>T), MTHFR2(1298A>C), HLA-G(−725C>G), VEGF(+405G>C), TNFα(−308A>G), AMHR(−482 A>G), PAI-1 (4 G/5 G), multiplex PCR assay was designed to genotype women undergoing ICSI program. We analyzed the overall study population (n=427) and a subgroup with homogeneous characteristics (n=112).

## Introduction

*In vitro* fertilization (IVF) is a complex, multistep process. Oocytes-containing follicles are collected after controlled ovarian hyperstimulation (COH) with follicle stimulating hormone (FSH). Some of the subsequently fertilized oocytes will be transferred to the uterus for implantation, whereas others may be cryopreserved for future implantation attempts (or destroyed if they are unlikely to survive cryopreservation). All these steps are critical for successful IVF.

The aim of COH is to safely obtain a high number of mature oocytes so that the most viable embryo can be selected for transfer. Both quantitative and qualitative factors in oocyte production have a high influence on the IVF outcome. The goal is to transfer a single embryo and thus reduce the risk of multiple pregnancies - the main complication of IVF [1].

The significant inter-individual variability to COH with FSH is one of the most challenging issues in IVF treatment. Although low responses are troublesome, high responses can trigger a serious medical condition - ovarian hyperstimulation syndrome (OHSS). Hence, the ability to predict an individual’s responses to COH would constitute a major advance in patient care. Although many hormonal and clinical parameters (such as baseline FSH [2], oestradiol [3], inhibin B [4] and anti-Mullerian hormone (AMH) levels [5], patient age [6] and the antral follicle count [7]) have been used to optimize COH, none of these markers have significant predictive value when considered alone [8], [9], However, predictive performance levels can be improved by considering combinations of these parameters [10].

Despite these advances in patient management, there is still a need to individualise and optimise stimulation protocols, reduce the likelihood of an extreme response and thus increase the probability of a live birth. A complementary strategy involves studying the pharmacogenetics of the COH response. Candidate genes should have a specific effect on the reproductive system and present single-nucleotide polymorphisms (SNPs) that affect gene expression or function.

**Table 1:** Allele frequencies of the studied SNPs in our population and in the NCBI database.

Candidate SNP	Allele frequencies			
	Allele	overall study population	Homogeneous subgroup	NCBI
AMH	A/C	654 (76%)/206 (24%)	159 (71%)/65 (29%)	82%18%
AMHR	AG	700 (82%)156 (18%)	176 (79%)48 (21%)	83%17%
BMP15	CG	193 (23%)663 (77%)	65 (29%)159 (71%)	23%77%
ESR1	AG	428 (52%)398 (48%)	112 (50%)112 (50%)	58%42%
ESR2	GA	520 (61%)336 (39%)	136 (61%)88 (39%)	62%38%
FSHR	AG	475 (56%)379 (44%)	136 (61%)88 (39%)	60%40%
HLA-G	CG	765 (90%)89 (10%)	197 (88%)27 (12%)	84%16%
MTHFR1	TC	216 (28%)563 (72%)	81 (36%)141 (63%)	31%69%
MTHFR2	AC	623 (73%)227 (27%)	160 (73%)60 (27%)	70%30%
p53	CG	272 (32%)582 (68%)	70 (31%)154 (69%)	26%74%
PAI	4 G4 G/5 G	445 (52%)409 (48%)	119 (53%)105 (47%)	50%50%
TNF	AG	112 (14%)676 (86%)	28 (12%)206 (88%)	17%83%
VEGF	GC	616 (78%)176 (22%)	156 (75%)52 (25%)	80%20%

Gene association studies have identified a number of SNPs (affecting gonadotrophin, steroid and TGFβ pathways, etc.) involved in the ovarian response. Most of them affect mRNA levels or the protein sequence and thus lead to quantitative or functional protein variations that may account for the observed inter-individual variability in the COH. The first SNP to be studied was the FSH receptor polymorphism Asn^680^Ser, which affects baseline FSH level and increases gonadotrophin requirements during COH [11], [12]. The ESR1 (−397 T>C) polymorphism was positively correlated with low oocyte retrieval after COH [13]. AMH (Ile^49^Ser) and AMHR polymorphisms (−482 A>G) have been associated with variations in oestradiol levels and may modulate FSH sensitivity [14]. More recently, it has been suggested that the COH outcome depends on combinations of genetic and environmental factors [15]. In support of this hypothesis, an oligo-SNP model (including FSHR: Asn^680^Ser, ESR1: −397 T>C, and ESR2:,+1730 A>G polymorphism) was reportedly associated with a low response to FSH during COH [13]. However, due to the small sample sizes and the heterogeneity of the studied populations, the impact of these polymorphisms requires further investigations.

Here, we sought to evaluate the impact of thirteen polymorphisms (all reportedly associated with variations in IVF results) in a population of women undergoing intracytoplasmic sperm injection (ICSI) program in our center. A multiplex PCR assay was developed and statistical analysis was performed to evaluate the SNPs individual and combined impacts on the COH outcome. We studied the overall study population of patients undergoing ICSI program and selected a subgroup that was homogeneous in terms of patient characteristics.

**Table 2:** Association of SNPs selected with ovarian response outcome.

Polymorphism	p value		p value after Holm’s correction	
	Overall study population	Homogeneous population	Overall study population	Homogeneous population
TNF	0.0955	0.9654	0.8595	1
PAI	0.4745	0.2068	1	1
MTHFR2	0.4177	0.3987	1	1
HLAG	0.1431	0.7065	1	1
BMP15	0.5599	0.0786	1	0.4680
ESR1	0.7381	0.6716	1	1
AMHR	0.4288	0.1247	1	0.4070
MTHFR1	0.1921	0.8440	1	1
AMH	0.1268	0.0747	1	0.6903
VEGF	0.6470	0.5295	1	1

**Table 3:** Multivariate analysis results in both populations.

PopulationGene	Overall studiedn=427	Homogenous n=112
ESR2	0.0511	Not included
p53	0.1685	Not included
FSHr680	0.1969	0.0002
Age	0.9017	0.3842
FSH level at J3	0.1409	Not included

## Results

In the overall study population, we observed a significant difference in the distribution of the oocyte number with age (p<0.0001) and FSH level (p=0.0044). However, in the homogeneous subgroup, only a slight difference in the distribution of the oocyte number according to age (p=0.0289) was noted.

The allele frequencies in the homogeneous subgroup and in the overall study population are listed in Table 1. The observed frequencies were similar to those quoted on the NCBI website (http://www.ncbi.nlm.nih.gov/snp).

After a univariate analysis, only three of the thirteen SNPs (FSHR (p.Asn^680^Ser, +2039 A>G); p53 (p.Arg^72^Pro, +215 C>G), and oestradiol receptor 2 (+1730 G>A) polymorphisms) appeared to be significantly associated with baseline characteristics and/or the number of mature oocytes. These polymorphisms were in Hardy-Weinberg equilibrium, with a 1% error interval.

The other polymorphisms (AMH, AMHR, BMP15, VEGF, MTHFR1, MTHFR2, ESR1, TNFα, HLA-G and PAI) did not appear to influence the number of mature oocytes collected (Table 2).

After applying Holm’s correction and a multivariate analysis (Table 3), the influence of p53 (p.Arg^72^Pro) was no longer statistically significant.

In a multilocus analysis of ESR2, p53, FSHR680 and AMH polymorphisms, only the FSHR Asn^680^Ser/AMH Ile^49^Ser combination was found to be associated with the number of mature oocytes after COH.

**Table 4:** The FSHR (Asn^680^Ser) polymorphism: baseline population characteristics, treatment parameters and ovarian response.

Candidate SNP (rs)	Population	Genotype	Number of women	Age (years)	Day-3 FSHlevel (IU/l)	Day-3 LH level (IU/l)	Amount of exogenous FSH required for ovulationinduction (IU)	Oestradiol level on the day of hCG administration(pg/ml)	Number of mature oocytes
**FSHR** **(rs6166)**	Overall studypopulation	Asn/Asn	142	30.1±12.6	6.6±3.1	4.2±2.0	2384±947	2197±941	7.1±3.9
		Asn/Ser	191	32.0±4.6a§	7.7±4.0b§	4.8±2.4c§	2386±976	2184±908	7.6±3.9
		Ser/Ser	94	32.0±5.3	7.3±2.8	4.8±2.2	2386±899	2172±1005	8.1±4.3^d,e,f^
		Asn/Ser + Ser/Ser	285	32.0±4.8^a$^	7.6±3.7^b$^	4.8±2.3^c$^	2207±951	2207±951	7.7±4.0
	Homogeneoussubgroup	Asn/Asn	45	30.3±3.5	6.5±1.7	4.0±1.5	2353±819	2128±802	7.2±4.0
		Asn/Ser	46	30.7±3.7	6.0±1.7	4.5±2.3	2287±748	2245±844	8.1±3.3
		Ser/Ser	21	30.7±3.0	6.8±1.5	4.2±2.2	2278±815	2271±962	9.8±4.6^g,h,i^
		Asn/Ser + Ser/Ser	67	30.7±3.5	6.3±1.7	4.4±2.2	2248±764	2253±875	8.6±3.8

The major allele was used as a reference.

a§ p value 0.0414 ^a$^p value=0.0241.

b§ p value=0. 0071, ^b$^p value=0.0126.

c§ p value c=0.0325, ^c$^p value=0.0207.

d,e,f Univariate p value=0.0047, Univariate p after Holm’s correction=NS, multivariate p value=NS.

g,h,I Univariate p value=0.0009, Univariate p after Holm’s correction=0.0225, multivariate p value=0.0002.

### The FSH Receptor Polymorphism (FSHR p.Asn^680^Ser, +2039 A>G) (Table 4)

In the overall study population, women with the Ser^680^ variant had significantly higher day-3 FSH and LH levels than women who were homozygous for the Asn^680^ variant (7.6±3.7 IU/l vs. 6.6±3.1 UI/l (p=0.0126) for FSH and 4.8±2.3 IU/l vs. 4.2±2.0 IU/l (p=0.0207) for LH, respectively). There was no such association with day-3 FSH or LH levels in the homogeneous subgroup (women under the age of 38 years old and with FSH levels <10 IU/l). The amount of FSH units administered and the oestradiol level on the day of HCG administration were similar for all genotypes in both the overall study population and the homogeneous subgroup.

**Figure 1:**
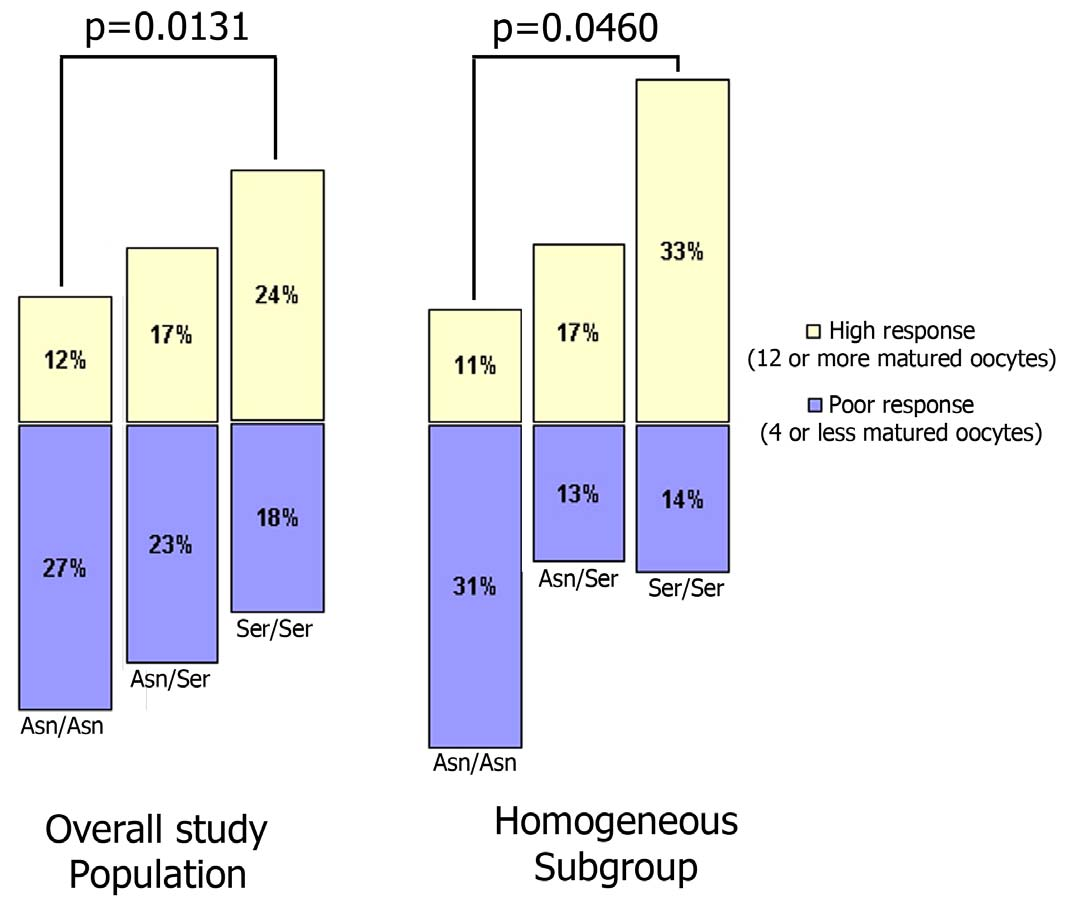
Poor and high response risks, according to FSHR^680^ polymorphism genotypes.

**Table 5:** The FSHR (Asn^680^Ser) polymorphism combined with the AMH (Ile^49^ Ser) polymorphism: baseline population characteristics, treatment parameters and ovarian response.

Candidate SNP (rs)	Population	Genotype FSHR (Asn680Ser)/AMH(Ile49 Ser)	Numberof women	Age (years)	Day-3 FSHlevel (IU/ml)	Day-3 LH level(IU/ml)	Amount of exogenous FSH required for ovulationinduction (IU)	Oestradiol level on the day of hCG administration(Pg/ml)	Number of mature oocytes
**FSHR/AMH**	Overall studypopulation	Asn^680^/Asn^680^//NNand/or NN/Ile^49^/Ile^49^	314	31.1±9.2	7.3±3.8	4.7±2.3	2395±966	2169±924	7.3±3.9
		Ser^680^/Ser^49^	14	32.0±3.3	8.6±4.2	4.7±2.5	2443±920	2100±972	10.3±5.5^a^
	Homogeneoussubgroup	Asn^680^/Asn^680^//NNand/or NN/Ile^49^/Ile^49^	82	30.6±3.5	6.4±1.8	4.4±2.0	2367±822	2149±877	7.9±3.9
		Ser^680^/Ser^49^	5	31.3±1.5	7.7±1.7	3.2±1.8	1770±619	2269±790	12.8±3.7^b^

NN: whatever the genotype.

The major allele was used as a reference.

a Univariate p value=0.0068.

b Univariate p value=0.0090.

Surprisingly, women who were homozygous for the Ser^680^ variant had a greater number of mature oocytes than women who were homozygous for the Asn^680^ variant, with averages of 8.1±4.3 vs. 7.1±3.9 oocytes (p=0.0047) in the overall study population and 9.8±4.6 vs. 7.2±4.0 oocytes (p =0.0009) in the homogeneous subgroup, respectively.

After applying Holm’s correction and a multivariate analysis, the FSHR Asn^680^Ser polymorphism was no longer statistically significant in the overall study population. However, it was still significantly correlated (p=0.0225) with the oocyte number in the homogeneous subgroup.

**Figure 2:**
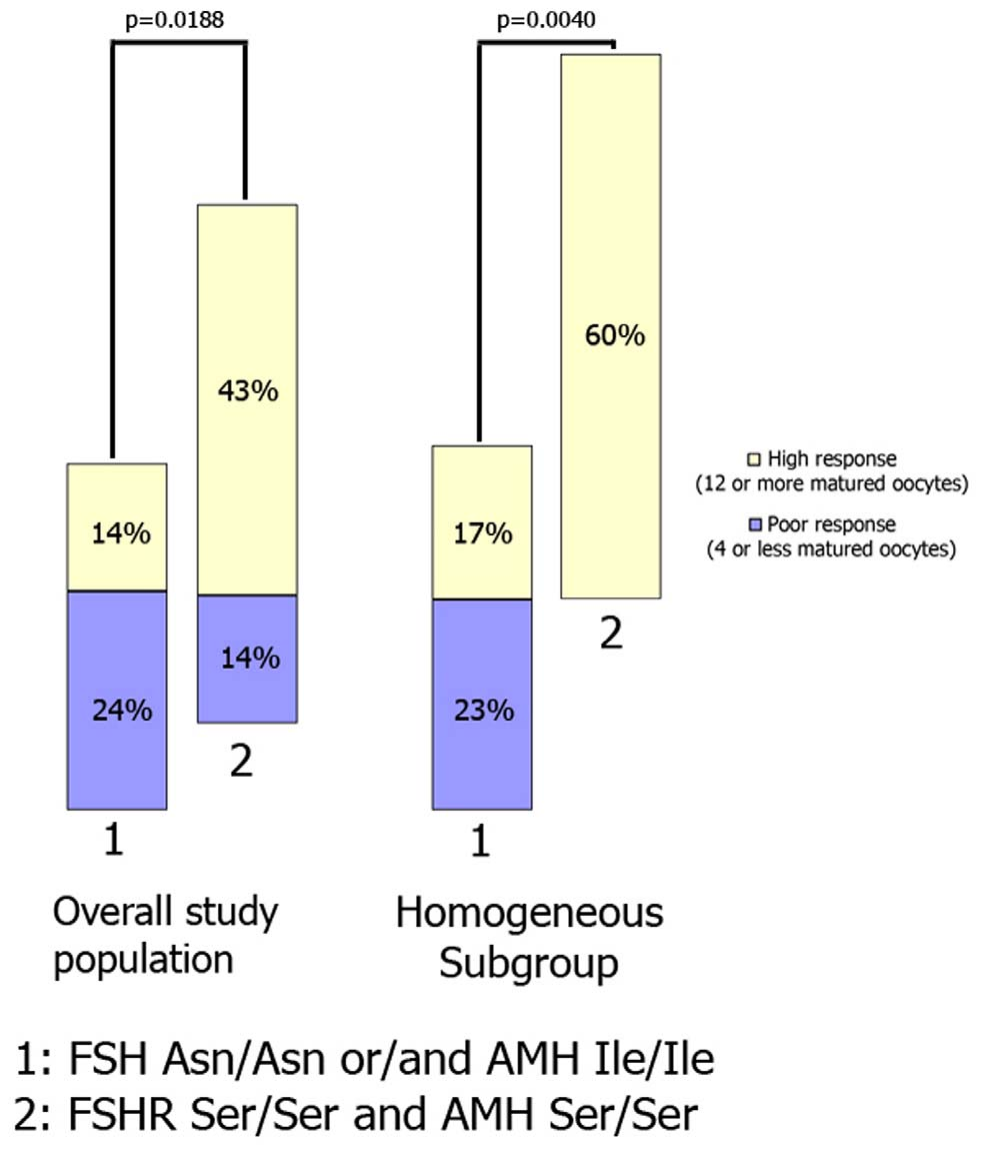
Poor and high response risks, according to FSHR^680^ and AMH^49^ polymorphism genotypes.

**Table 6:** The ESR2 (+1730 G>A) polymorphism: baseline population characteristics, treatment c parameters and ovarian response.

Candidate SNP (rs)	Population	Genotypes	Women number	Age (years)	Day-3 FSHlevel (IU/ml)	Day-3 LH level(IU/ml)	Amount of exogenous FSH required for ovulationinduction (IU)	Oestradiol level on the day of hCG administration(Pg/ml)	Number of mature oocytes
**ESR2 (rs4986938)**	Overall studyPopulation	GG	158	30.9±9.3	7.2±3.7	4.7±2.3	2282±847	2396±1015	8.1±4.2
		GA	204	30.8±6.1	7.4±3.3	4.5±2.1	2457±999	2067±726a§	7.2±4.0^b,c,d^
		AA	66	31.1±3.2	7.2±3.9	4.7±2.3	2390±1000	2049±915a||−	7.3±3.6
		AA+AG	270	31.6±7.6	7.4±3.5	4.6±2.2	2440±847	2063±872a$	7.2±3.9^e^
	Homogeneoussubgroup	GG	40	30.6±3.2	6.8±1.4	3.7±1.4	2375±752	2299±936	7.9±3.6
		GA	56	30.8±3.1	6.3±1.7	4.8±2.3f§	2145±744^g§^	2238±844	8.2±4.4
		AA	16	30.3±3.2	6.4±1.8	3.7±1.6	2706±879g||−	1849±494	8.0±3.1
		AA+AG	72	30.6±3.6	6.4±1.6	4.6±2.2^f$^	2276±807	2148±791	8.1±4.1

The major allele was used as a reference.

a§ p value=0.0016,

a||− p value=0.0155,

a$ p value 0.0007.

b,c,d Univariate p value=0.0017, Univariate p after Holm’s correction=0.0425, multivariate p value=NS.

e p value=0.0314.

f§ p value=0.0095, ^f$^p value=0.0346.

g||− p value AG compared to AA=0.0150.

**Table 7:** The p53 (Arg^72^Pro) polymorphism: baseline population characteristics, treatment parameters and ovarian response.

Candidate SNP (rs)	Population	Genotype	Women number	Age (years)	Day-3 FSHlevel (IU/ml)	Day-3 LH level(IU/ml)	Amount of exogenous FSH required for ovulationinduction (IU)	Oestradiol level on the day of hCG administration(Pg/ml)	Number of mature oocytes
**p53 (rs1045222)**	Overall studypopulation	Arg/Arg	214	31.5±8.0	7.4±3.1	4.8±2.4	2361±978	2162±885	7.8±4.3
		Arg/Pro	154	31.6±5.1	7.1±3.5	4.4±1.9	2387±960	2169±1006	7.2±3.9
		Pro/Pro	59	30.3±3.9	7.4±4.8	4.3±2.2	2416±781	2322±952	7.0±3.3
		Arg/Pro + Pro/Pro	213	31.5±8.0	7.2±3.9	4.4±2.0	2248±997	2401±916	7.1±3.7
	Homogeneoussubgroup	Arg/Arg	60	30.1±3.0	6.6±1.8	4.4±2.1	2175±702	2204±760	8.8±3.9
		Arg/Pro	34	31.0±4.1	5.9±1.6	4.5±1.9	2446±862	2132±984	7.0±4.1^a,b,c^
		Pro/Pro	16	31.1±3.7	6.3±1.6	3.3±1.7	2476±836	2335±862	7.7±3.0
		Arg/Pro + Pro/Pro	50	31.1±3.9	6.1±1.6	4.1±1.9	2198±942	2457±845	7.2±3.8^d^

The major allele was used as a reference.

a,b,c Univariate p value=0.0048, Univariate p after Holm’s correction=NS, multivariate p value=NS.

d p value=0.0451.

Moreover, in the overall study population, we observed that women who were homozygous for the Ser^680^ variant were less likely to have had a low response than women who were homozygous for Asn^680^ (18% vs. 27%, respectively). Likewise, women who were homozygous for the Ser^680^ variant were more likely to have had a high response than women who were homozygous for Asn^680^ (24% vs. 12%, respectively) (p=0.0131). These observations were also confirmed in the homogeneous subgroup (p=0.046) (Figure 1).

### The Combination of FSHR and AMH Polymorphisms (Table 5)

Within both the overall study population and the homogeneous subgroup, the AMH Ile^49^ Ser polymorphism was not associated with any clinical or hormonal parameters or the number of oocytes retrieved. However, in both populations, women who were homozygous for both the FSHR Ser^680^ variant and AMH Ser^49^ variant yielded more mature oocytes than women who were homozygous for FSHR Asn^680^ and/or homozygous for AMH Ile^49^ with 10.3±5.5 and 7.3±3.9 mature oocytes (p=0.0068) in overall study population and 12.8±3.7 vs. 7.9±3.9 mature oocytes (p=0.009) in the homogenous subgroup.

In the overall study population, we observed that women who were homozygous for the FSHR Ser^680^ and AMH Ser^49^ variants were less likely to have had a low response than women who were homozygous for FSHR Asn^680^ and/or homozygous for AMH Ile^49^ (14% vs. 24%, respectively). Similarly, women who were homozygous for the FSHR Ser^680^ and AMH Ser^49^ variants were more likely to have a high response than women who were homozygous for FSHR Asn^680^ and/or homozygous for AMH Ile^49^ (43% vs. 14%, respectively) (p=0.0188). These observations were confirmed in the homogeneous subgroup (p=0.004) (Figure 2).

### The Oestradiol Receptor 2 Polymorphism (ESR2+1730 G>A) (Table 6)

In the overall study population, there was no association between this polymorphism and the day-3 serum levels of LH and FSH. However, in the homogeneous subgroup, an elevated LH serum level was observed for heterozygous women, with mean LH levels of 3.7±1.4, 4.8±2.3 and 3.7±1.6 IU/l for the GG, GA and AA genotypes, respectively. Day-3 E2 and FSH levels did not vary with genotype in the homogeneous subgroup.

The amount of FSH administered was not associated with the polymorphism in the overall study population. However, the mean E2 level on the day of hCG administration was higher in women who were homozygous for the G allele (2396±1015 pg/ml) than in heterozygous women and women who were homozygous for the A allele (2067±726, p=0.0016 and 2049±915 pg/ml, p=0.0155 respectively).

In the overall study population, women who were homozygous for the G allele had a greater mean number of mature oocytes than (i) women who were heterozygous for the A allele (8.1±4.2 vs. 7.2±4.0, respectively; p=0.0017) or (ii) group of women who were homozygous or heterozygous for the A allele (8.1±4.2 vs. 7.2±3.9, respectively; p =0.0314).

The results differed in the homogeneous subgroup, where the number of mature oocytes was not associated with the polymorphism. However, women who were homozygous for the A allele required more exogenous FSH (2706±879 IU) than women who were homozygous or heterozygous for the G allele (2375±752 IU and 2145±744 IU respectively) to produce a similar number of oocytes.

In the overall study population, the ESR2 polymorphism was still significantly correlated with the oocyte number after applying Holm’s correction (p=0.0425) but not after a multivariate analysis (p=0.0511). In both populations, there were no relationships between this polymorphism and the likelihood of a low or high response.

### The p53 Gene Polymorphism (p.Arg^72^Pro, +215 C>G) (Table 7)

In both populations, there were no genotype-related differences in terms of age, day-3 hormone levels, the amount of FSH required for ovulation induction and the E2 level on the day of HCG administration.

In the overall study population, the number of mature oocytes obtained was not significantly associated with the genotype).

In the homogeneous subgroup, women who were homozygous for the Arg^72^ variant had a greater number of oocytes than (i) women who were heterozygous for the Pro^72^ variant (8.8±3.9 vs. 7.0±3.3, respectively; p=0.0048) and (ii) group of women who were homozygous and heterozygous for Pro^72^ variant (8.8±3.9 vs. 7.2±3.3, respectively; p =0.0451). However, this difference was no longer significant after applying Holm’s correction and a multivariate analysis.

**Figure 3:**
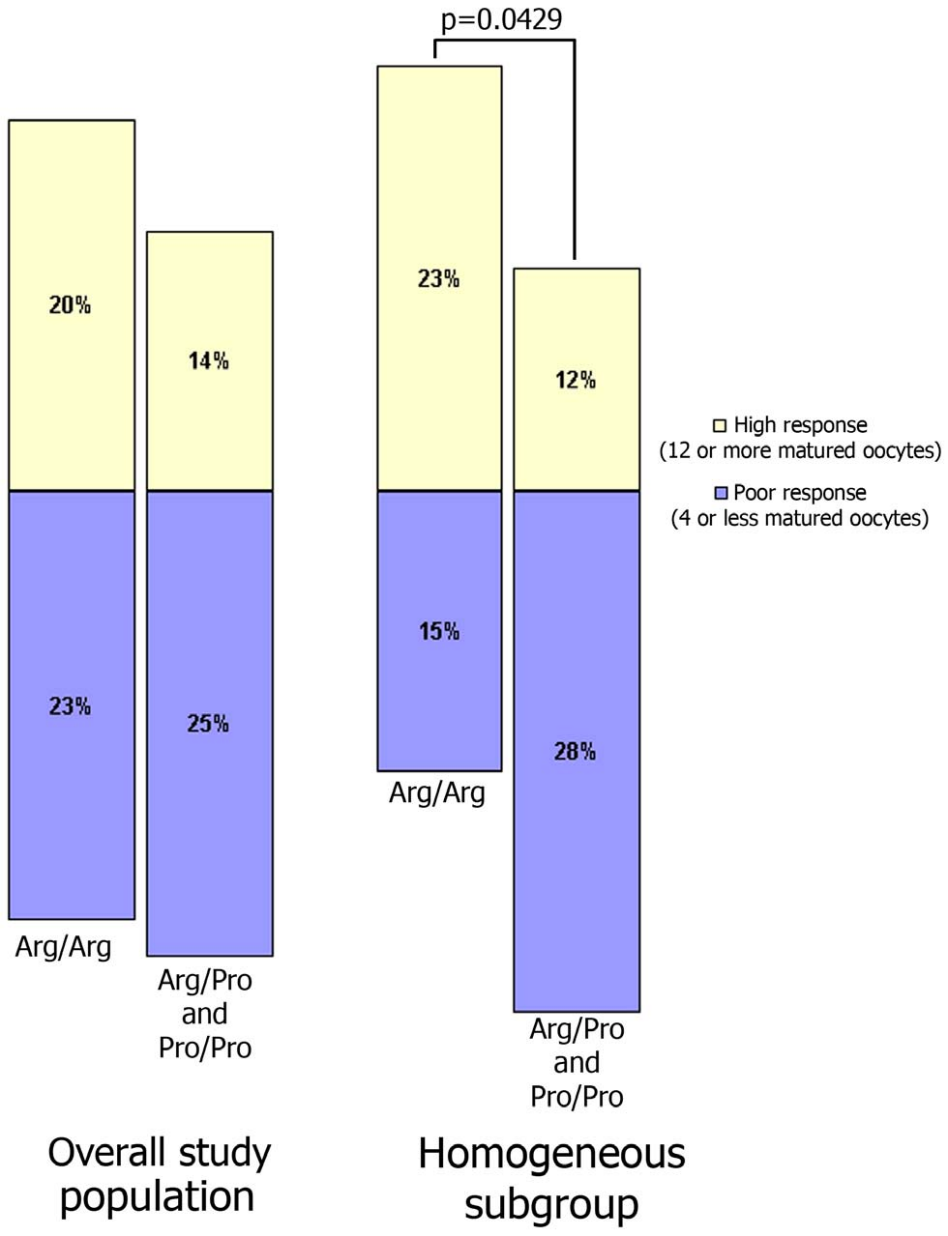
Poor and high response risks, according to p53^72^ polymorphism genotypes.

Moreover, in the homogenous subgroup, we observed that women who were homozygous for Arg^72^ were less likely to have a low response than women who were heterozygous or homozygous for the Pro^72^ allele (15% vs. 28%, respectively). Accordingly, the women who were homozygous for Arg^72^ were more likely to show a high response than those who were heterozygous or homozygous for Pro^72^ allele (23% vs. 12%, respectively; p=0.0429) (Figure 3).

**Table 8:** Women characteristics.

Women characteristics		Overall study population	Homogeneous subgroup
Patients number (n=)		427	112
Age (years)		31,2±9.55	30.6±3.53
**Hormonal profile**	**Day-3 E2 level(IU/ml)**	46.27±26.15	46.89±28.17
	Day-3 LH level (IU/ml)	4.64±2.77	4.32±2.02
	Day-3 FSH level (IU/ml)	7.02±2.44	6.40±1.74
ICSI indication	IVF failure	28.6%	0%
	Male infertility	71.4%	100%
Menstruel cycle	Normal	77.6%	100%
	Abnormal	22.4%	0%
Ovulation	Normal	21.7%	100%
	Abnormal	78.3%	0%
Ethnic origin	Caucasian	80%	100%
	Others	20%	0%

## Discussion

Of the thirteen polymorphisms studied here, only three SNPs (in the genes coding for FSHR, ESR2 and p53) and one SNP combination (FSHR Asn^680^Ser/AMH Ile^49^Ser) appeared to be significantly associated with the number of mature oocytes retrieved after COH. To improve our analysis, we applied Holm’s correction for p-values and performed a multivariate analysis to evaluate the polymorphisms’ respective impacts on the women’s IVF results.

The polymorphisms studied here have been previously shown to affect the response in some but not all studies.

We did not genotype FSHr307 in the current study because it is in near-complete linkage disequilibrium with FSHr680 [16]. In the present study, we investigated gene-environment interactions. Both genetic variants and environmental factors (as age, ovulation and length of cycle) have a significant influence on the ovarian response to gonadotrophins.

We showed that some effects are not apparent in the unselected ICSI population and clearly highlights the care that must be taken when comparing these studies. For example, FSH and LH levels on day 3 were not dependant on the FSH receptor polymorphism but a difference was found in the total unselected ICSI population. Thus, differences between studies might reflect the heterogeneity of included patients. In the homogeneous subgroup, the woman’s age and FSH level had less impact on the number of oocytes retrieved and so clearer conclusion could be drawn in this respect - even though the sample size was very small.

### The FSH Receptor Polymorphism (FSHR p.Asn^680^Ser, FSHR 2039 A>G)

In the homogeneous subgroup, there was no relationship between the genotype on one hand and the day-3 FSH level or age on the other - mainly because an FSH level below to 10 IU/l and age under 38 were criteria for inclusion in this subgroup.

In contrast, in the overall study population, we found that a significantly higher day-3 serum FSH level was associated with the FSHR Ser^680^ variant. The increased age might explain the increased day 3 FSH. These data indicate that the polymorphism has no effect on young patients but might interfere when the patients are getting older.

The genotype did not appear to be associated with the amount of exogenous FSH. The attending gynaecologists were blinded to the genotype at the time of prescription. Similarly, there was no FSHR genotype-related difference in the E2 level on the day of hCG administration.

As described in the Results section, the women who were homozygous for the FSHR Ser^680^ variant were less likely to have been low responders and more likely to have been high responders. These results were confirmed in the overall study population. Furthermore, the statistical significance after Holm’s correction and a multivariate analysis confirmed the impact of the FSHr680 genotype on the ovarian response to rFSH in IVF cycles.

Several previous studies have sought a correlation between the FSHR polymorphism and the outcome of the ovarian response but yielded discordant results.

In contrast with our present results, it has been reported that FSHR Asn^680^Ser is associated with a low E2 level during ovarian stimulation. To achieve similar oestradiol peak levels, homozygous FSHR Ser^680^ women were found to need more exogenous FSH than women with FSHR Asn^680^ [11], [12], [17]. Indeed, other researchers have suggested that women with the FSHR Ser^680^ polymorphism have a higher ovarian threshold for FSH and thus a longer follicular cycle. The FSHR Ser^680^ polymorphism has also been linked to lower sensitivity to the action of FSH [18].

Other studies have not found any association between FSHR Ser^680^ polymorphism and various baseline hormone levels or the amount of exogenous FSH required for ovarian stimulation [19]–[22]. More recently, it was reported that women who were homozygous for the FSHR Asn^680^ polymorphism needed higher amounts of exogenous FSH and tended to need more stimulation days [23]. This observation is concordant with our present study, in which women who were homozygous for FSHR Asn^680^ had fewer mature oocytes than women homozygous for FSHR Ser^680^ (despite the administration of similar amounts of exogenous FSH).

In other studies, women who were homozygous for the FSHR Ser^680^ polymorphism were more at risk of a high response and iatrogenic OHSS after similar ovarian stimulation [22], [24]. Only the occurrence of moderately intense OHSS was correlated with the FSHR Ser ^680^ polymorphism [24]. Our results were similar, with an increased likelihood of a high response (≥12 mature oocytes) for women who were homozygous for the FSHR Ser^680^ polymorphism. The occurrence of iatrogenic hyperstimulation is probably linked to the hypersensitivity to FSH; this is quite unexpected because the Ser^680^ polymorphism has previously been correlated with low sensitivity to FSH [11].

There is currently no evidence of an effect of the FSHR genotype on hormone binding characteristics or cAMP or inositol phosphate production following FSH stimulation. It is also possible that the FSHR Asn^680^Ser polymorphism only has a direct impact on the ovarian gonadotrophin response and oocyte recruitment when it is combined with other polymorphisms.

This hypothesis was strengthened by the results of our study, since the association between the FSHR polymorphism and the number of mature oocytes appeared to be higher when combined with the AMH Ile^49^Ser polymorphism (which is thought to slightly alter the biological activity of AMH [25]).

In both the overall study population and the homogeneous subgroup, the AMH Ile^49^Ser polymorphism alone was not significantly associated with baseline day-3 hormone levels or the number of mature oocytes recovered after COH. Our results agree with previous data in this respect (Hanevik et al. 2010).

In both the overall study population and the homogeneous subgroup, we found that women who were homozygous for both the FSHR Ser^680^ and the AMH Ser^49^ alleles had a significantly greater mean number of mature oocyte numbers than women who were homozygous for FSHR Asn^680^ and/or homozygous for AMH Ile^49^– even though the various groups received similar amounts of exogenous FSH. A genotype-related difference was also present in the overall study population and the homogeneous subgroup when we compared the likelihood of belonging to the subgroups formed according to the number of oocytes. An increased likelihood of a high response was observed for women who were homozygous for both the FSHR Ser^680^ and the AMH Ser^49^ alleles, when compared with women who were homozygous for FSHR Asn^680^ and/or homozygous for AMH Ile^49^.

It has been shown that AMH-knockout mice display fast, high-quality primordial follicle recruitment and have a more pronounced response than the wild type in the presence of high serum FSH concentrations. In view of these data, AMH inhibits primordial follicle growth *in vitro* [26]–[28] and attenuates sensitivity to FSH. Conversely, follicles are more responsive to FSH in the absence of AMH [29], [30].

These data are in agreement with our results, which suggest that AMH and FSH polymorphisms might improve the recruitment of primordial follicles. Our findings also suggest that the combination of AMH and FSHR polymorphisms may have potential value as a marker for the ovarian response in women undergoing IVF treatment but due to sample size this finding needs to be confirmed.

**Table 9:** Gene polymorphisms reportedly involved in IVF outcomes.

Gene name	Protein name	Protein function	Variant Name	Association with ovarian response		Association with embryo implantation	
				Positive association	Negative association	Positive association	Negative association
***AMH***	Anti-Mullerian hormone	Hormone	+146 G/T (p.Ile^49^Ser)	[14], [25]	[50]		
***AMHR2***	Anti-Mullerian hormone type IIreceptor	Hormone receptor	–482 A/G	[14], [25]	[50]		
***BMP15***	Bone morphogenic protein 15	Oocyte and follicle development	−9 C/G	[51]			
***ESR1***	Oestrogen receptor 1	Hormone receptor	−397 T/C	[13], [52], [53]			
***ESR2***	Oestrogen receptor 2	Hormone receptor	+1730 A/G	[13], [31], [52], [53]			
***FSHR***	Follicle-stimulating hormonereceptor	Hormone receptor	+2039 A>G (p.Asn^680^Ser)	[11]–[13], [17]–[22], [24], [54]–[56];	[57]	[54], [57]	
***HLA-G***	Major histocompatibilitycomplex class I, G	Antigen processing and presentation	–725 C/G			[58]	
***MTHFR1***	Methylenetetrahydrofolatereductase	Folate metabolism	+677 C/T (p.Ala^222^Val)	[59]	[60]	[61]	[62]
***MTHFR2***	Methylenetetrahydrofolatereductase	Folate metabolism	+1298 A/C (p.Glu^429^Ala)	[60]		[63]	
***P 53***	Tumour protein p53	Apoptosis and DNA reparation	+215 C/G (p.Arg^72^Pro)	[48]		[44]	
***PAI-1***	Plasminogen activatorinhibitor-1	Tissue development and coagulation	−675 (4 G/5 G)			[64]	
***TNF***	Tumour necrosis factor alpha	Pro-inflammatory cytokine	−308 A/G			[65]	
***VEGF***	Vascular endothelial growthfactor	Vascular permeability and angiogenesis	+405 G/C			[66]	

### The Oestrogen Receptor β Gene Polymorphism (ESR2+1730 G>A)

In the overall study population, women who were heterozygous or homozygous for the A allele had a significantly greater number of mature oocytes than those who were homozygous for the G allele, despite receiving similar amounts of exogenous FSH. Statistical impact of ESR2+1730 genotype on ovarian response to rFSH in IVF cycle was confirmed using Holm’s correction but not using multivariate analysis. This discrepancy is probably due to sample size, limited for multivariate analysis.

No difference was observed in the homogeneous subgroup; women who were homozygous for the A allele needed to receive more exogenous FSH than women who were heterozygous for the A allele (2706±879 IU and 2145±744 IU, respectively; p=0.0150) to achieve adequate oocyte maturation and obtain a similar number of oocytes. Heterozygous women received similar amounts of exogenous FSH when compared with women who were homozygous for the G allele but had a higher day-3 LH level; which may contribute to best final adequate oocytes maturation. There were no genotype-related differences in the likelihood of belonging to the low-response or high-response groups.

In view of previous reports [31] associating ESR2 polymorphism with ovulatory dysfunction, we believe that our fertility-based inclusion criteria for the homogeneous subgroup (i.e. excluding women with known causes of infertility) can explain why the ESR2 polymorphism was not associated with the nature of the ovarian response.

Although oestrogen’s action is mediated by the ESR1 and ESR2 receptors, the latter predominates in the ovary [32], [33]. ESR2 stimulates early folliculogenesis, decreases follicular atresia and stimulates late follicular growth [34] by inducing the action of FSH. In turn, FSH promotes granulosa cell proliferation. ESR2 action may thus explain the synergistic effect of oestrogen and FSH on the number of FSH receptors in granulosa cells (resulting in follicular growth and maturation [35]). However, our data did not reveal an association between the FSHR p.Asn^680^Ser and ESR2+1730 G>A polymorphisms on ovarian response.

ESR2 knockout mice have inefficient ovulation efficiency and produce few oocytes. This is mainly due to a defect within ovarian tissue in general [36], [37] and inability of E2 to exert its effect on the granulosa cells in maturing follicles in particular. Furthermore, it has been shown that ESR2 had no effect on the serum concentration of pituitary reproductive hormones [38]. Our results are in agreement with ESR2-knockout mouse data, since the mRNA of the ESR2+1730 A variant folds differently and is expressed less [39]. Furthermore, it has also been shown that follicles with low oestrogen level had low-quality, apoptotic oocytes [40], [41], which could reduce the number of mature oocytes.

Hence, our results suggest that the ESR2+1730 G>A polymorphism modulates the IVF outcome by affecting the number of mature oocytes.

**Table 10:** Primer design for the selected SNPs.

Gene name	Referencesequence	Primer (5′-3′)			PCR product size
AMH	rs10407022	F1 F2 R	CACAGAGGCTCTTGTGGG**C** CACAGAGGCTCTTGTGGG**A** GATAGGGGTCTGTCCTGCAC	FAM HEX	247
AMHR	rs2002555	F1 F2 R	CCTTCCTCTGCCCAAGC**A** CCTTCCTCTGCCCAAGC**G** CCAGCTGAGAACCCAGTGAT	FAM HEX	207
BMP15	rs3810682	F1 F2 R	GAGGAGGACCATCTTGAAAG**G** GAGGAGGACCATCTTGAAAG**C** ATGAGGCAACTTTGGTCCAG	FAM HEX	197
ESR1	rs2234693	F1 F2 R	GAGTTCCAAATGTCCCAGC**T** GAGTTCCAAATGTCCCAGC**C** GGGGAAATTGTTTATTGCAAAC	FAM HEX	234
ESR2	rs4986938	F1 F2 R	GGCCCACAGAGGTCACA**G** GGCCCACAGAGGTCACA**A** CTTCCTCACACCGACTCCTG	FAM HEX	157
FSHR	rs6166	F1 F2 R	GACAAGTATGTAAGTGGAACCA**T** GACAAGTATGTAAGTGGAACCA**C** TGTTTCACCCCATCAACTC	HEX FAM	224
HLA-G		F1 F2 R	TGAAACTTAAGAGCTTTGTGAGTC**C** TGAAACTTAAGAGCTTTGTGAGTC**G** AGTTGTGCCTGAGTGCATGA	FAM HEX	191
MTHFR1	rs1801133	F1 F2 R	GAAGGTGTCTGCGGGAG**C** GAAGGTGTCTGCGGGAG**T** AGAACTCAGCGAACTCAGCA	FAM HEX	238
MTHFR2	rs1801131	F1 F2 R	GAGGAGCTGACCAGTGAAG**C** GAGGAGCTGACCAGTGAAG**A** ACAGGATGGGGAAGTCACAG	HEX FAM	178
p53	rs10425222	F1 F2 R	CAGAGGCTGCTCCC**C** CAGAGGCTGCTCCC**G** GACTTGGCTGTCCCAGAATG	FAM HEX	163
PAI	rs1799889	F1 F2 R	TCA**GGGG**CACAGAGAGAGTC TCA**GGGGG**CACAGAGAGAGTC CAGCCACGTGATTGTCTAGG	FAM HEX	148149
TNF	rs1800629	F1 F2 R	ATAGGTTTTGAGGGGCATG**A** ATAGGTTTTGAGGGGCATG**G** GAGTCTCCGGGTCAGAATGA	FAM HEX	184
VEGF	rs2010963	F1 F2 R	CTCACTTTGCCCCTGTC**G** CTCACTTTGCCCCTGTC**C** GAGGCGCAGCGGTTAG	HEX FAM	351

### The p53 Gene Polymorphism (p. Arg^72^Pro, +215 G>C)

In the overall study population and in the homogeneous subgroup, the FSH treatment did not vary as a function of the p53 polymorphism. However, for the homogeneous subgroup, we observed that women who were homozygous for the Arg^72^ allele had a significantly higher mean number of retrieved oocytes than women who were heterozygous or homozygous for the Pro^72^ polymorphism. As this result was no longer statistically significant after Holm’s correction and a multivariate analysis, these data should be considered with a degree of caution. Further analyses are required to confirm or repudiate this finding.

Moreover, the genotype p53 Arg^72^ appeared to increase the likelihood of a high response and decrease the likelihood of a low response in the homogeneous subgroup.

The p53 tumour suppressor protein plays a crucial role in maintaining genomic stability in somatic cells. It has been shown that small changes in the level and/or activity of p53 can alter its functional efficiency. In *Drosophila* and *C. elegans*, p53 protein is most commonly expressed in germ cells, where it eliminates defective gametes and, consequently, defective offspring from the population [42], [43].

The first report of an impact of p53 on fertility found a high association between the Pro^72^ polymorphism and recurrent implantation failure [44]. It has been further suggested that p53 regulates female reproduction and blastocyst implantation through transcriptional up-regulation of uterine leukocyte inhibitory factor (LIF), which is an important factor in implantation. Elevated endometrial LIF levels are observed at the time of implantation in fertile women [45]. Women with unexplained infertility have lower LIF levels than fertile women do [46], [47].

In agreement with our present results, it has been reported that the Pro^72^ polymorphism is highly associated with decreased pregnancy rates after fresh IVF, via an ovarian mechanism [48]. Even though functional impact of p53 on oogenesis has not yet been investigated, one can hypothesize that low p53 activity is associated with greater DNA damage during folliculogenesis and oogenesis. Although genetic predispositions must always be confirmed in larger series, the p53 Arg^72^Pro polymorphism appears to have a significant impact on the ovarian response in women undergoing IVF treatment.

In conclusion, our objective was to determine a genetic profile suitable for use prior to the IVF protocol, in order to adjust the amount of prescribed FSH. We genotyped women after participation in an IVF protocol and investigated the potential usefulness of genetic testing for predicting the COH response. On the basis of literature reports, we identified thirteen polymorphisms that may impact ovarian function. Using a univariate statistical analysis, we found that three of the latter polymorphisms (in the genes coding for the FSHR, ESR2 and p53) and one combination of polymorphisms (FSHr680/AMH) were significantly associated with the number of mature oocytes retrieved after COH. After Holm’s correction and a multivariate analysis, p53 was no longer statistically significant.

There is a need for clinical studies in which the amount of FSH given to patients is modulated according to the genotype - especially for women who are genetically predisposed to low or high responses to COH.

**Figure 4:**
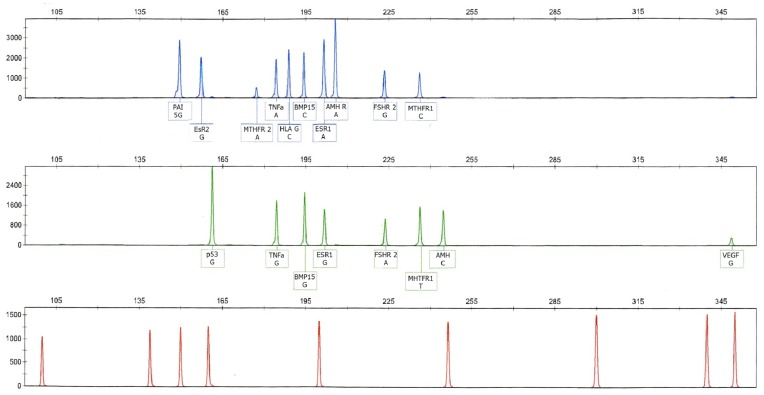
Electrophoregram profile with the 13 polymorphisms genotyped. The first and second lines show alleles fluorescently tagged with FAM and Hex dyes, respectively. The last electropherogram is the RO×500 size standard.

## Materials and Methods

### Subject Population

The study was approved by an independent ethics committee (the *Comité Consultatif de Protection des Personnes dans la Recherche Biomédicale*; project reference: 01032) and was performed in accordance with the tenets of the Declaration of Helsinki. All the women provided their prior, written, informed consent to participation.

We included a total of 427 women undergoing an initial ICSI procedure with oocyte retrieval and embryo transfer for severe male infertility. Before the ICSI procedure, the patients had been extensive evaluated in terms of their personal and family medical history, clinical and serological status, hysterosalpingography, day-three hormonal profile (FSH, luteinizing hormone and estrogen) and karyotype. The exclusion criteria included, prior chemotherapy, unilateral ovariectomy, maternal diethylstilbene treatment, an abnormal karyotype or any identified genetic abnormalities.

To lessen the impact of age and FSH level on our results, we then selected a Caucasian subgroup (n=112) that was homogeneous in terms of age and the absence of known aetiological factors for female infertility. The criteria for this subgroup were as follows: Caucasian origin, normal karyotype, age under 38, a day-three serum FSH level below 10 IU/l and the combination of a long GnRH agonist desensitization protocol and treatment with recombinant FSH for COH. The exclusion criteria included uterine malformation, grade 3 or 4 endometriosis, polycystic ovary syndrome (PCOS). Clinical and demographic characteristics were described in Table 8.

Ovarian follicle stimulation was performed with recombinant FSH (GonalF® from Merck Serono or Puregon® from Organon-Schering Plough) and monitored by estrogen measurements and transvaginal ultrasound from day 5 onwards. Ovulation was induced with 10,000 IU of hCG or recombinant hCG. Transvaginal, ultrasound-guided follicle aspiration was performed 35 hours later and maturity of oocytes with a single polar body was evaluated after hyaluronidase treatment.

The number of mature oocytes obtained after ovarian hyperstimulation was analysed by genotype. In order to evaluate the association between the SNPs and the average number of oocytes in response to COH, we divided the population into three categories: low responders (4 oocytes or less), normal responders (between 5 and 11 oocytes) and high responders (12 oocytes or more).

### DNA Preparation

For each patient, a blood sample was collected for DNA analysis. Genomic DNA was extracted using the Wizard® Genomic DNA Purification Kit (Promega, Southampton, UK), according to the manufacturer’s protocol. Genotyping was performed after the IVF procedures had been completed.

### SNPs Selected for Genotyping

We selected thirteen SNPs that reportedly impact the ovarian response and/or embryo implantation in women in IVF programmes (Table 9):

### Primer Design

For each of these thirteen selected SNPs, an allele-specific PCR assay was developed. Primer pairs were designed using Primer3 online software (www.ncbi.nlm) (Table 10). Each primer set was carefully designed for compatibility with a multiplex PCR assay, with a (i) products between 150–350 base pairs that can be identified on capillary electrophoresis and (ii) a melting temperature (T_m_) close to 60±2°C. Each allele-specific forward or reverse primer was marked with either 6-carboxyfluorescein (FAM, blue colour) or hexachlorocarboxyfluorescein (HEX, green colour) and synthesized by Eurogentec (Serain, Liege, Belgium). After receipt, primers were diluted to an appropriate concentration for PCR assays in 10 mM Tris buffer and stored at −20°C until use.

### Allele-specific PCR Validation

The 20 µl PCR reaction mixture (QIAGEN Hilden, Germany) contained 2 µl of extracted DNA, 0.2 µM of each primer. A Silver 96-Well GeneAmp 2700 PCR System was used for DNA amplification. A denaturation step was first performed (15 min at 95°C) followed by 24 PCR cycles (denaturation: 30 seconds at 94°C; annealing: 90 seconds at 65°C; extension: 60 seconds at 72°C) and a final extension phase (30 min at 60°C). All experiments were repeated twice.

### Multiplex PCR Conditions

Multiplex PCR was carried out in 20 µl reaction volumes, containing 2 µl of extracted DNA containing 0.2 µM of all primers Thermal cycling was performed using the conditions described above. All experiments were also repeated twice.

### Genotyping

PCR products were diluted 1∶10 in sterile water, and 1 µL of this dilution was added to 20 µL of a mixture containing 19.5 µL Formamide® (Applied Biosystems, Foster City, California, USA) and 0.5 µl RO×500 size standard (Applied Biosystems) in 96-well PCR plates. The samples were denatured for 3 min at 95°C.

Multiplex PCR products were separated by capillary electrophoresis using an ABI Prism 3100 genetic analyzer (Applied Biosystems). Allelic call was performed using the Genemapper® ID v.3.1 software (Applied Biosystem) (Figure 4).

### Statistics

Statistical analysis was performed in three steps:

#### 1. Univariate analysis

For age, the day-3 serum levels of LH and FSH, the amounts of exogenous FSH and the oestradiol level on the day of hCG administration, means were compared in an analysis of variance. The threshold for statistical significance was set to 5%.

For the oocyte number, the means were compared using generalized linear models (GLMs) supported by the GENMOD procedure (SAS Institute Inc., Cary, NC, USA), with the hypothesis of a Poisson distribution for the response variable.

For each genotype, the respective proportions with a high response (≥12 mature oocytes) and a low response (≤4 mature oocytes) were compared in a Stat view program (SAS institute) using Chi-2 and Fisher tests.

To take into account multiple testing for the polymorphisms, the familywise error rate was adjusted with the sequential Bonferroni-Holm procedure [49]. Deviations from the Hardy-Weinberg equilibrium were assessed by means of a Chi-2 test.

#### 2. Multivariate analysis

We performed a multivariate analysis with all polymorphisms and putative confounding clinical factors (age, FSH level, cycle length and ease of ovulation). All polymorphisms found to be significant at p≤0.30 after Holm’s correction were introduced as covariates into GLM models. Any suitable, first-order interactions derived from these factors were also added as covariates. Final models were obtained using backward selection of variables, with the likelihood ratio as the selection criterion. Variables with p>0.05 and <0.10 were kept in the final models.

#### 3. Multilocus analysis

Lastly, in order to better identify polymorphism interactions, a multilocus analysis was performed with GLM models. All polymorphisms found to be significant at p≤0.075 in the univariate analysis (without Holm correction) were introduced into the multilocus analysis; this enabled us to decrease the number of independent covariates in the models. Backward selection of variables was performed as above, and variables with p>0.05 and <0.10 were kept in the final models.
